# Smart Pipes—Instrumented Water Pipes, Can This Be Made a Reality?

**DOI:** 10.3390/s110807455

**Published:** 2011-07-27

**Authors:** Nicole Metje, David N. Chapman, David Cheneler, Michael Ward, Andrew M. Thomas

**Affiliations:** 1 School of Civil Engineering, College of Engineering and Physical Science, University of Birmingham, Edgbaston, Birmingham, B15 2TT, UK; E-Mails: d.n.chapman@bham.ac.uk (D.N.C.); andrewmarkthomas@yahoo.com (A.M.T.); 2 School of Mechanical Engineering, College of Engineering and Physical Science, University of Birmingham, Edgbaston, Birmingham, B15 2TT, UK; E-Mails: d.cheneler@bham.ac.uk (D.C.); m.c.ward@bham.ac.uk (M.W.)

**Keywords:** smart pipes, intelligent water distribution networks, MEMS, smart technology, structural monitoring

## Abstract

Several millions of kilometres of pipes and cables are buried beneath our streets in the UK. As they are not visible and easily accessible, the monitoring of their integrity as well as the quality of their contents is a challenge. Any information of these properties aids the utility owners in their planning and management of their maintenance regime. Traditionally, expensive and very localised sensors are used to provide irregular measurements of these properties. In order to have a complete picture of the utility network, cheaper sensors need to be investigated which would allow large numbers of small sensors to be incorporated into (or near to) the pipe leading to so-called smart pipes. This paper focuses on a novel trial where a short section of a prototype smart pipe was buried using mainly off-the-shelf sensors and communication elements. The challenges of such a burial are presented together with the limitations of the sensor system. Results from the sensors were obtained during and after burial indicating that off-the-shelf sensors can be used in a smart pipes system although further refinements are necessary in order to miniaturise these sensors. The key challenges identified were the powering of these sensors and the communication of the data to the operator using a range of different methods.

## 1. Introduction

The buried utility pipeline infrastructure is the primary asset of a network owner and operator. Its maintenance, both in terms of attention to its physical components and to its operational state, is of the utmost importance to minimise disruption to the infrastructure and the population in its locality. Consequently, routine monitoring of the performance of the infrastructure (both physically and operationally) is essential to the planning of its maintenance. In an ideal situation, a monitoring system, introduced into the utility/pipeline infrastructure, would warn of impending failure. Existing systems broadly achieve the above aims, but they lack accuracy due to the limitations of the technology involved. In addition, they are expensive to introduce and may cause water quality problems. It should be noted that utilities and their maintenance should not be treated in isolation since utilities are generally laid beneath roads, and hence maintenance and emergency repair operations often seriously disrupt road users.

Another issue with existing systems is that currently, water distribution systems are only monitored at discrete points in the distribution network. Due to the costs of these measuring stations, it is not possible to monitor the whole network and hence it can sometimes be difficult to identify local problems with the system, such as corrosion failures or leaks, until they are either reported by the customer or are visible at surface level. If the network could be monitored more extensively, for example via a large number of miniaturised sensors incorporated into the pipe material, within coatings on pipelines or within the ground around pipes, it would allow a more proactive, and ultimately cost-effective, management regime of the whole network. As current technology is not available for a distributed monitoring network, alternatives have to be investigated. Micro-Electro-Mechanical System (MEMS) technology has shown its potential in many different applications: aerospace, automotive, home entertainment and biomedical, to deliver small, cost-effective sensors [1–3]. It has therefore been identified as an ideal technology for the wide, distributed pipe network system, where large numbers of low-cost sensors will be required, as one of the key advantages of MEMS technology is that large numbers of sensors can be manufactured at very low cost. With suitable electronic communication systems, either built into a pipe-traversing pig (pipeline inspection gauge—pig) or located at regular intervals along a pipe, which can detect the sensor signals, and with appropriate transmission and interpretation, such so-called *smart* pipes become possible. The MEMS sensors need not be sophisticated, but due to their large numbers, will allow for the comprehensive monitoring of the whole network.

This paper primarily concentrates on initial results of a proof-of-concept prototype smart pipe system which was built and buried in the summer of 2009 on the University of Birmingham, UK campus. The focus was to show that such a system is theoretically feasible, even though much research is still required into individual components of the system. At the same time, initial results of the research carried out on communication, power and miniaturisation of the sensors are provided.

## 2. Background

A number of techniques have been developed over the years to investigate the condition of water pipes and in particular to detect leaks. A detailed summary of some of these non-destructive techniques is given in [4,5]. These techniques include intelligent pigs using magnetic flux leakage or remote eddy current techniques, and the Sahara™ equipment using acoustic methods. More recently alternative techniques for locating leaks in water pipes have come to market, for example the Smart Ball™ [6]. This involves inserting a ‘ball’ containing acoustic sensors and data acquisition capabilities into the water or wastewater pipe. It travels along the pipe collecting data and once extracted from the pipe the data are analysed. More increasingly Ground Penetrating Radar (GPR) is used not only to detect pipelines, but also to determine defects and leaks [7]. However, many of these techniques rely of personnel going out into the field and investigating specific sections of pipe and so can be time consuming and labour intensive. There are commercial sensors available that can be placed at discrete locations along a pipeline network (often at valve locations) to do spot checks on, for example water quality [8]. However, sensors for monitoring pipe deterioration or leak detection are not routinely installed for water pipes due to lack of suitable systems, cost and the inaccessibility of buried water pipes.

Over the past decade or so there have been major developments in the areas of wireless sensor devices, power sources and sensor systems within engineering. Some of these developments are briefly described below, as these are important if smart pipes are to become a reality.

Wireless sensor devices (motes) can be combined to form networks which can be used to monitor the condition of buried utility systems. There are many issues that need to be addressed when developing a wireless infrastructure monitoring system. These issues stem from the fact that these systems require many different technologies. Each device will in general contain a power source, a communication link and sensors to measure, for example, strain, temperature, vibration or chemical content, as well as other circuitry [9].

Often the wireless component in current wireless sensor networks is a single point to point link to a base station where the communication cable has been replaced with a radio system [9]. In order to extend the network to monitor infrastructure on a large scale it is necessary to use the wireless sensor devices as an integral part of the communication network. They will forward each others data and act as bridges to the operator, while tolerating individual failures and changing patterns of ad-hoc communication.

A number of wireless communication interfaces and protocols are being developed in order to accommodate multiple transducer connectivity such as WiFi, Bluetooth and Zigbee [10,11]. As well as fixed networks, these technologies can be combined to form Mobile Ad-hoc NETworks (MANET), a collection of mobile computing devices co-operating to form a dynamic network with no fixed infrastructure [12]. In a MANET, computing nodes themselves become an integral part of the communications infrastructure, communicating with each other over vast areas using multi-hop routing algorithms.

Sensors are the “eyes, ears, noses and taste buds” of these networks. Current wireless sensor networks employ micro technologies to interact with their environment. Miniaturisation of sensors and incorporation with integrated circuitry (IC) technology has been possible due to various developments in surface mounted devices, System in Package (SiP) and System on Chip technology which allow complex circuits to be produced in a very condensed package [13].

As wireless communication systems using radio frequencies tend to require several mW when transmitting, reduced to μW on average using a sleep cycle, and as modern batteries can generate at best 1 J/mm^3^—existing motes will have a life expectancy of around 14 h operation using a cubic mm battery [14]. Micro fuel-cells, whose development is being driven by the mobile phone industry [15], are predicted to store around 10 J/mm^3^, increasing the mote’s life tenfold [16]. It is reasonable to assume that advances in sensor, communication and processing design will reduce the power needed, however this still only provides a finite life expectancy. It is therefore likely that future networks will require some means of scavenging and storing energy from their surroundings. This may be in the form of vibration powered energy scavengers [17,18], thermogenerators [19] or surface mounted solar panels [20]. A number of excellent reviews, which discuss the issues involved in wireless sensor networks already exist, for example [21–23].

Examples of large scale deployments of the technology include the Smart Pebble, so called because it is the size of a typical piece of rock aggregate, which has been used to monitor chlorine levels in concrete structures [24]. The device is based on RFID technology and the chlorine sensor can be interrogated and powered remotely. Also, a joint venture between Purdue University, Notre Dame University and the EmNet Corporation has resulted in a wireless sensor network for a sewage system [25]. The system uses a citywide network of 105 manhole-mounted sensors and “smart valves” to automatically control storm runoff. The PipeNet project was designed to detect leaks in water pipe networks [26]. It was based on the Intel Mote Sensor Node which uses Bluetooth to transmit data from the various sensors. The system used 6 V, 12 Ah batteries which were shown to last around 55 days using the systems sleep mode when not transmitting. This can be compared to the Golden Gate project where Crossbow Mode hardware was used. In this case, the battery used supplied 18 Ah, but only lasted 23 days due to the high data transfer rates used [27]. In addition, the Intelligent Trench project makes use of radio-frequency identification (RFID) technology and GPS asset marking for locating buried utilities [28].

## 3. Methodology and Results

### 3.1. The Smart Pipe Demonstrator Unit

**Figure 1.**
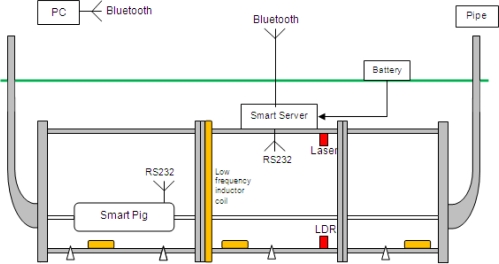
Schematic arrangement of the smart pipe demonstrator unit [29].

For the development of a smart pipe system, issues related to communication, sensor development and integration within and around the pipe as well as the power requirements of such a system need to be investigated. A range of sensors and communication elements were incorporated into a smart pipe demonstrator unit utilising mainly off-the-shelf MEMS components. The aim was to demonstrate the concept and show how the individual elements could work together. Different modes of communication were investigated, with the signals travelling through the ground, along the pipe and to a so called Smart Server capable of amplifying the signal. Figure 1 shows the schematic of the smart pipe demonstrator unit. It is expected that any sensor system for water pipes will require a combination of sensors to detect a range of potential problems.

This demonstrator unit comprised a number of communication systems, which are described later and a range of sensors including piezoelectric transducers, force sensors, light detection circuits, two-axis accelerometers and randomly distributed temperature sensors (all hard wired to the Smart Server). The data gathered from these sensors were transmitted from the Smart Server via a Bluetooth module situated on the ground surface.

**Figure 2.**
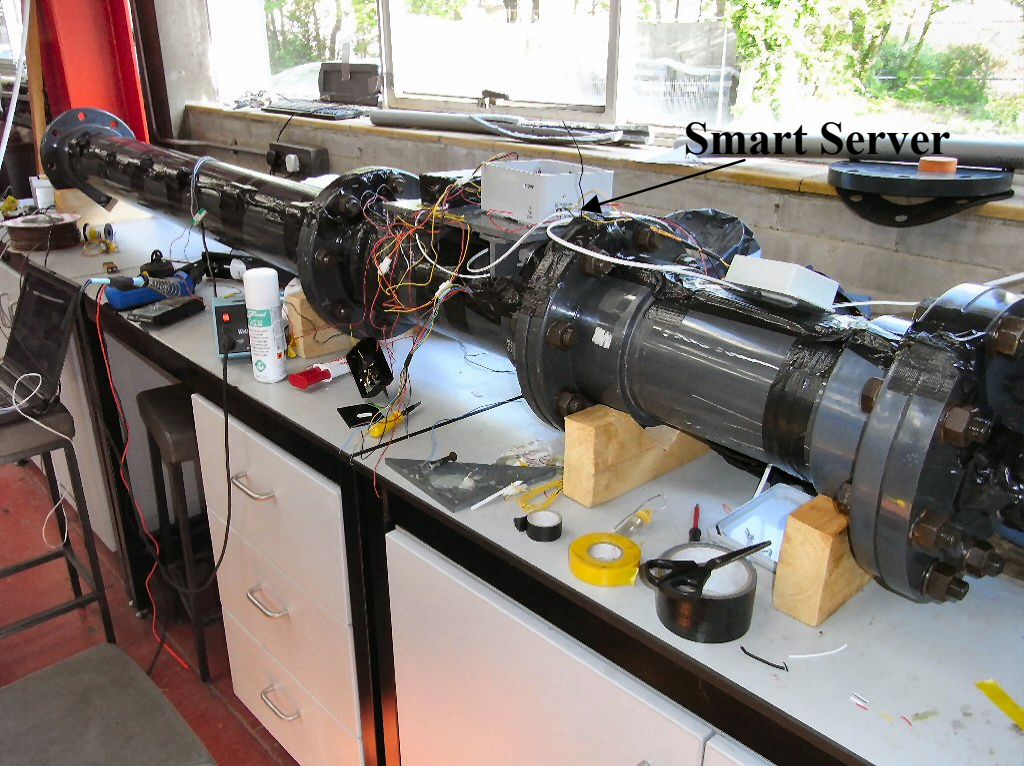
Smart pipe demonstrator unit before burial.

Figure 2 shows the smart pipe demonstrator unit before burial in the laboratory. The unit consisted of lengths of PVC pipe with an internal diameter of 150 mm.

**Figure 3.**
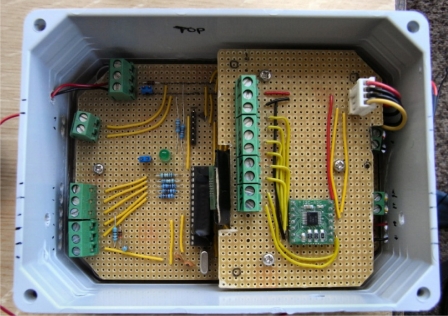
Prototype Smart Server [29].

A smart sensing system which can collect sensor signals and transmit these to a Smart Server (Figure 3) was developed. The idea behind the Smart Server is that the individual sensors (due to their small size and power requirements) would only be capable of transmitting information over a short distance, while the Smart Server could house a system capable of storing and transmitting the data a longer distance along the pipe or through the ground. This function could be utilised in a practical smart pipe system, where units with more power could be located at certain intervals along the pipe capable of storing and then transmitting data as required. It is envisaged that this could be developed so that the Smart Server transmits the data on a regular basis, possibly once a month, but it would also have on-board analysis software that could recognise a change in data and, if it is outside a set range of trigger values, would transmit these data immediately and thus highlighting a problem.

**Figure 4.**
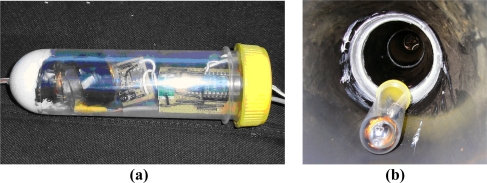
Smart Pig device **(a)** prior to insertion into the smart pipe demonstrator unit and **(b)** inside the pipe [29].

An alternative approach was the use of a Smart Pig, which incorporated sensors, electronics and signal tranmission components. It could traverse along a pipe and collect, store and transmit data from the randomly distributed sensors within and around the pipe. This could either be used on its own or in combination with the Smart Servers. The Smart Pig, for example, could collect data from the sensors and then upload these data to the Smart Server as it passes where upon these data could be transmitted from the Smart Server out of the pipe network. Figure 4 shows the Smart Pig used in the demonstrator unit.

The Smart Pig was made from a plastic container with a diameter of 50 mm and a length of 150 mm. It contained an inductor loop connected to a function generator on the surface. This was used in order to produce signals for low frequency communication. It also contained a microcontroller and RS232 system for high frequency communication. The Smart Pig was connected to a wire in order to feed it through the pipe and to supply the signal from the surface based function generator. Although feeding a pig through a real pipe network is not ideal, it showed the principle of using such a system. Such a system could be useful as an interim technology whilst powering issues of the sensors are overcome as this could obtain data from passive sensors.

The demonstrator unit incorporated wireless and passive systems which were tested in parallel with other forms of communication in order to indentify their range and power requirements. The tape around the pipe in Figure 2 indicates the position of the inductive communication coils and temperature sensors. The smart pipe incorporated two inductor loops, comprising of copper wire wound a number of times around the pipe. One was connected directly to the Smart Server, which measured the voltage induced in the coil due to nearby low frequency communication systems. The other coil was situated 500 mm along the pipe and was connected to a surface based function generator.

The temperature sensors were LM35DZ (National Semiconductor, USA) centigrade scale temperature sensors and simply chosen to demonstrate the concept of collecting data from multiple sensors and transmitting these data. Local rises in temperature can be used to indicate leaks and temperature sensors can also be used to warn of extreme temperature changes in the pipe due to ambient weather conditions. In the UK, the extremely cold weather in December 2010 over a relatively long period (for the UK) caused a higher than average number of buried water pipes to fail [30].

The piezoelectric transducers and force sensors were situated by the Smart Server between the flanges of two connected sections of pipe and were used to detect changes in stress at the joints. This type of sensor is important as a large proportion of pipe leakage and damage is created when a pipe is disturbed and the stress in the soil surrounding the pipe changes, for example, due to adjacent excavations when other utlity providers repair their own services or by seasonal shrink-swell volume changes in clay soils [31].

The light detection circuit comprised a visible red laser module and light dependent resistor (LDR) positioned on opposite sides of the pipe (see Figure 1), and were axially aligned to reduce the effects of refraction in water. The light detection circuit formed a simple water turbidity dectector, which could be used for water quality monitoring or as an indication of leaks. Previous research at Birmingham [32] has shown that leaking pressurized water pipes buried in fine grained soil cause some of the soil to be passed into the pipe and create greater turbidity in the water.

The two-axis accelerometer was fixed inside the Smart Server itself and used in a similar way to piezoelectric transducers and force sensors to detect distrubance to the pipe. Examples of the results obtained from the temperature sensors, the piezoelectric transducers the force sensors and the light detection circuit are presented in Section 3.2.

**Figure 5.**
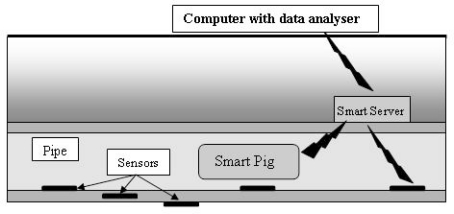
Schematic arrangement of possible Smart Pipe systems involving a Smart Pig and Smart Server arrangement, and a Smart Server and sensors.

Together, the Smart Pig and Smart Server would be the essential components of a smart pipe system for occasional analysis (in combination with passively powered sensors). For a system that is capable of regular monitoring, *i.e.*, without requiring access to insert a Pig, a combination of powered sensors, Smart Servers and a surface-level collection and interpretation system would be required. These options are shown schematically in Figure 5.

The following sections provide details of the smart pipe demonstrator unit burial and a selection of results obtained from this field experiment. It should be noted that there was also considerable research conducted in the laboratory on issues related to communication, MEMS sensor design and interpretating data from randomly distributed sensors using finite element analyses, however this is not discussed in this paper but more details can be found in [29].

#### The Smart Pipe Burial

The smart pipe demonstrator system was buried within a shallow trench (*c.* 800 mm deep) on the University of Birmingham campus. In order to ensure that the sensors and electronics remained operational, and to gather some initial data on the motion sensors which were affected by the burial works, the Smart Server remained powered by a 12 V battery during the burial and sensor outputs were monitored throughout the burial and backfilling processes.

**Figure 6.**
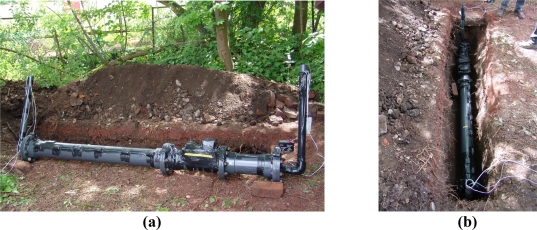
The smart pipe demonstrator unit **(a)** prior to placement in the 0.8 m deep trench and **(b)** placed in the trench ready for backfilling [29].

The ground conditions consisted of a sandy soil with many larger pieces of broken concrete and stone, and appeared to comprise made-ground, elevated above an adjacent stream behind the fence (Figure 6(a)). The smart pipe demonstrator unit was then taken to the burial location and lowered gently into place by hand (Figure 6). It was then backfilled ‘gently’ using the excavated material to prevent damage to the sensors and electronics.

Example data gathered and the lessons learnt from the burial of the demonstrator unit are presented in the following sections.

### 3.2. Sensors

The sensors used on the smart pipe demonstrator were off-the-shelf components based on MEMS technology chosen to represent the variety of data that are available. The types of sensors used include piezoelectric transducers, force sensors, light detection circuits, two-axis accelerometers and randomly distributed temperature sensors. As these commercial sensors were not designed specifically for integration into buried infrastructure, there are advantages in developing and miniaturising these sensors. The issues and results arising from a preliminary investigation into miniaturisation is discussed in Section 3.5.

**Figure 7.**
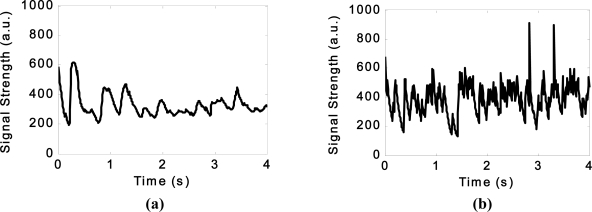
The vibrations detected by the piezoelectric transducers **(a)** as the pipe is being lowered into the trench and **(b)** as the response of the buried pipe to a revving car parked at the ground surface.

**Figure 8.**
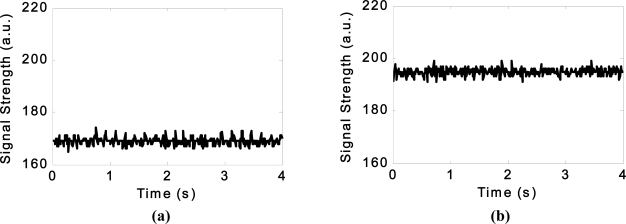
The response of the force transducer **(a)** as the pipe is being lowered into the trench and **(b)** after the backfill was placed.

**Figure 9.**
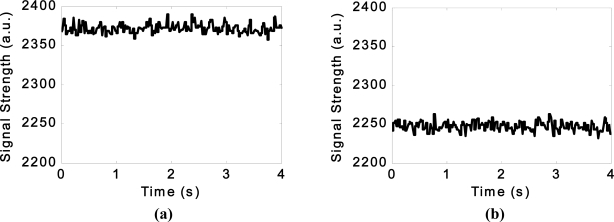
The response of the dual-axis accelerometer as a car is revved whilst parked on top of the buried pipe **(a)** x-axis, **(b)** y-axis.

The results from several of these sensors are shown below, however further information can be found in [29]. An example of the data obtained from the piezoelectric transducer, force transducer and dual-axis accelerometer within the smart pipe demonstrator unit as it is subjected to various types of excitation is shown in Figures 7 to 9. The additional load due to the backfill is registered by the force transducer as shown in Figure 8(b).

**Figure 10.**
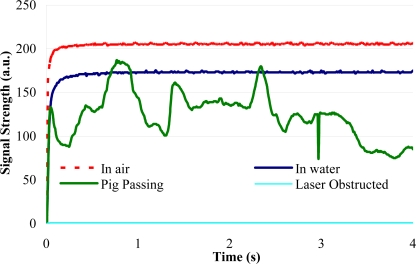
Variations in detected light intensity by the LDR under various conditions.

In addition, the light and temperature sensors provided some promising results. Figure 10 shows the variations in detected light intensity by the LDR in air and water, when the Smart Pig went past and when the LDR was obstructed.

Note how the attenuation of the light in water has caused the signal to drop significantly suggesting that changes in intensity may also identify air entrainment as well as suspended solids in the water. The trace related to the pig passing is of interest as it shows the change of light intensity as the wire connected to the pig obstructs the light to different degrees as it moves along the pipe.

**Figure 11.**
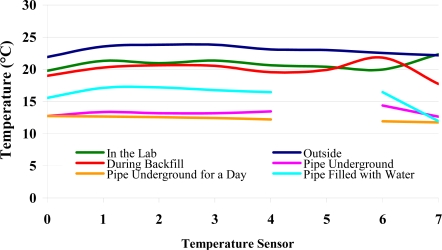
Temperature data collected from the various temperature sensors along the pipe when exposed to different conditions.

Figure 11 shows the temperature data collected from the various temperature sensors along the pipe when exposed to different conditions. Not only does it show that all but one (sensor 5) of the sensors survived the burial and were providing useful data after burial, it also shows that they did react to the pipe being flooded by warm water after it was buried. It also indicates that the variations along the pipe were small and that the temperature varied only small amounts once buried.

The data outputs from the demonstrator unit indicated that even very inexpensive sensors can provide useful data. In particular, the piezoelectric transducers incorporated into a flange on the demonstrator unit showed significant sensitivity at less than $1 US per unit, and can potentially provide data on external and/or internal stress changes on the pipe, while also being used to harvest power from say vehicles moving along the road above the pipe.

### 3.3. Communication

Although the sensors incorporated in the demonstrator unit were mainly hard-wired, significant research was conducted on the feasibility of wireless communication. The challenges with regards to communication are the power requirements and the media through which the signals travel. The transmission of the signals particularly through wet media is significantly affected by signal attenuation, especially at high frequencies [33]. Similarly, soils vary widely in their electromagnetic properties due to variations in mineralogy, water content and density. This means while transmissions through sandy soils may cause manageable attenuation, receiving them through soils with significant clay contents (which are prevalent over much of the UK) will potentially involve coping with severe attenuation that may prevent the use of traditional high frequency communication methods.

Therefore, wireless communication between, and from, these sensors present a key challenge. The scenario of a buried pipe encapsulates a number of communication systems (see Figure 5), and the signals they transmit and receive will travel through different media, each with different properties. For these reasons, options for communications between sensors, from sensors to a Smart Pig, from the sensors to Smart Servers, between Smart Servers and from Smart Servers to the ground surface level can be expected to be different. After careful consideration of the different types of communication envisaged, it became clear that there may be a potential need for both low (kilohertz) and high (hundreds of megahertz) frequency communication systems. The potential applications of the different systems are detailed in Table 1.

**Figure 12.**
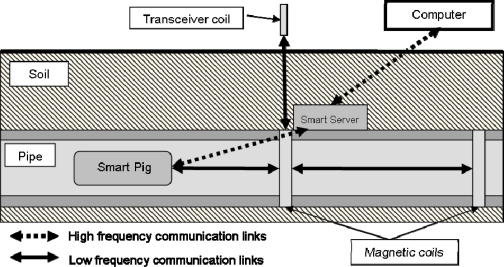
Schematic of the different communication links for the smart pipe demonstrator.

In the smart pipe demonstrator, two methods of collecting and controlling the collection of data were considered: one in the form of continuous collection, transmission, receipt and interpretation of data from sensors, with data collection Smart Servers and transmission coils situated at regular positions along the length of the pipe; and the second in the form of a commonly-used pig device with data collection and transmission components built-in (Smart Pig). The different communication links, are shown in Figure 12. The high frequency communication systems were all digital in nature. The Smart Server communicated to the computer using a Bluetooth module located at the soil surface. The Smart Pig, as well as using a low frequency system, also communicated to the Smart Server using a high frequency (433 MHz) superheterodyne transceiver system. There was no appreciable decrease in signal strength for this system as the Pig was moved along the short distance of the pipe.

A selection of results from the low frequency communication systems, which were based on inductive coupling, is given below.

**Figure 13.**
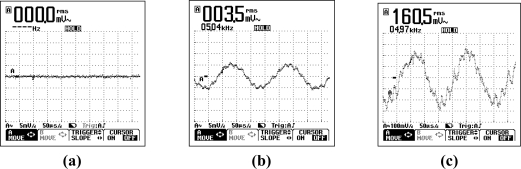
Communication along the pipe **(a)** without amplification, **(b)** with amplification and **(c)** with pre-amplification [29].

*Communication along the pipe*: Tests included attempts to transmit and receive signals over a wide range of frequencies between approximately 1 kHz and 10 MHz, all of which provided usable received signals. As an illustration, Figure 13(a) shows an oscilloscope trace at 5 kHz, illustrating that the received signal requires amplification as otherwise it cannot be detected. In Figure 13(b), the same signal is increased significantly in amplitude, but is still small and would be expected to reduce sharply if the coil spacing is increased. In Figure 13(c), a pre-amplifier is used and indicates that suitable amplification is important in the detection of these signals, as the diameter and number of wire turns of the coil are considered fixed by practical aspects such as pipe geometry. However, overall the test was considered to show successfully that low frequency transmission along the pipe is possible.

*Communication through the ground to the surface*: Initial tests for sensor to surface transmission involved the use of a small search coil, as illustrated in Figure 12. This allowed for the testing of two issues: communications through the soil to the surface using simple inductive coupling methods and secondly, using the same coil used for communication through water to locate the Smart Server when the pipe is buried.

**Figure 14.**
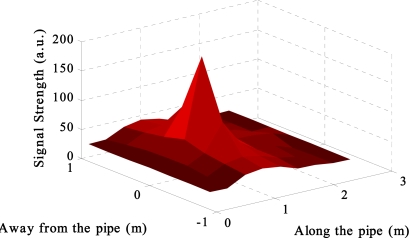
Signal strength detected by the Smart Server whilst buried.

By monitoring the response from the Smart Server, the strength of the signal was mapped out by moving the transmitting coil intermittent distances in the region of the pipe. The results of the mapping (shown in Figure 14) clearly show that it is possible to use this method to locate the Smart Server, perhaps even to communicate to the Smart Server. It is also interesting to note how it seems that it is also possible to determine the orientation of the pipe by denoting the signal strength parallel and perpendicular to the axis of the receiving coil.

**Figure 15.**
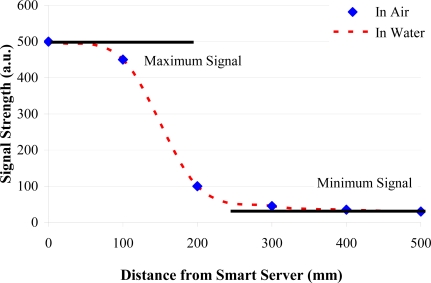
The signal strength as detected by the Smart Server as the Pig is moved along the pipe in air and in water.

*Communication between Smart Pig and Smart Server*: The low frequency inductive communication system between the Smart Pig and the Smart Server was tested by connecting a frequency function generator to the coil inside the Pig. The detector was a large coil connected directly to the Smart Server. The frequency of the function generator was set to the resonant frequency of the system in air (∼13.8 MHz) to achieve the maximum signal strength. The data in Figure 15 show how the strength of signal varies with distance when 1.5 Vpp was applied to the coil in the Pig. It should be noted that because the magnetic permeability of water is the same as that of air for low signal strengths, the range of communication through the two media is practically identical.

While the range is quite low (around 300 mm), signal strength is easily increased by increasing the voltage being applied to the coil in the Smart Pig. It is important to note that, at a certain distance, the signal strength becomes comparable to the noise in the system. Further increases in the distance between the Smart Pig and Smart Server cannot then be detected, as there is, essentially, no observable signal. It is still possible, however, that digital processing may allow communications to be achieved in this case. Although, if the Smart Pig is used to upload data collected from the sensors as it passes along the pipe to Smart Servers as it reaches them, the required transmission distance could be relatively small.

### 3.4. Power

The specification of the final system will be a complex compromise between three critical system design parameters: power consumption of individual sensors, data rate (how often and accurately a sensor reading is measured and recorded) and the communication range along which each sensor is required to transmit its data. The components utilised within the smart pipe demonstrator unit consumed approximately 150 mA at 12 V. This represents a power supply requirement of 1.8 W. To put this into context, a typical AA battery has a capacity of perhaps 2 Ah, and so at the same rate of power consumption would be expected to last less than two hours. Clearly, such a battery lifetime is not suitable for a real system, it is also inconceivable that any energy scavenging system could supply such a significant amount of power.

In any real system, there are considerable energy savings that can be made indicating that a smart pipe system is indeed achievable. The first major energy saving comes from the fact that the time constant of the physical asset is very long. If we ignore sudden catastrophic events, most asset changes will take place over days, weeks or even years. Thus, it is not necessary to run a sensor system continuously. Rather, we may only need to switch it on once a day, perhaps even less frequently. Additionally, the sensors can measure and transmit data very quickly, and need to be activated for only 100 ms to complete and transmit a measurement. If a sensor with an instantaneous power requirement of 1 W needs to only be activated for 100 ms per day, this represents a continuous power consumption of approximately only 1.0 μW. Under these conditions, a continuous power drain of 1.0 μW would give an AA battery a lifetime in excess of 100 years! The continuous power consumption of a more realistic system is likely to be much less than 1.0 W and is more likely to be set by the RF (radio frequency) communication system, which is typically 5.0 mW for a transmitted range of approximately 10 m, using ZigBee technology [11]. Thus, this leads to real systems (sensor, microprocessor and RF communications) with continuous power consumptions of perhaps just 20 mW, which, when used for just 100 ms per day, would give an averaged continuous power requirement of just 20 nW.

In trying to evaluate the possible power levels that may be available from energy scavenging devices, it is useful to consider the kinetic energy (½ mν^2^, where m is mass and ν is velocity) of water passing a point. For a pipe of 100 mm in diameter, and with water flowing at 1 m/s the kinetic power is of the order of Watts. Thus, it seems not unreasonable to expect to be able to generate the tens of nW required.

The piezo discs, mentioned previously, and located within the flanges in the demonstrator unit, showed considerable sensitivity and could offer the opportunity of harvesting energy from passing traffic and from vibrations associated with the water flowing within the pipe.

The above indicates that, while no suitable energy scavenging devices exist, it is not unreasonable to believe that they will become available, making the smart pipe an achievable reality in the medium term. The estimated power supply requirements do not appear to present any insurmountable problem.

### 3.5. Miniaturisation

**Figure 16.**
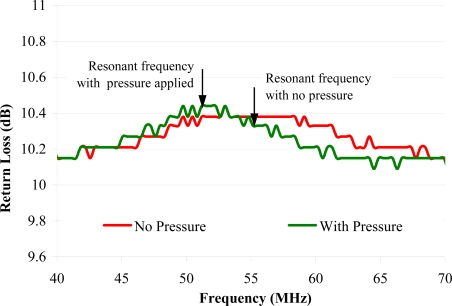
Change in resonant frequency of a sensor due to changes in pressure as measured through the pipe wall.

As mentioned before, there are advantages in miniturising the sensors. The main advantage is that smaller sensors could be integrated into the pipe wall, without affecting its integrity, in some manner that is more convenient to the utility owners, provided that the sensors can be powered and communicated with effectively. To demonstrate a possible method of powering and communicating with MEMS sensors, a simple compliant parallel plate capacitor with a capacitance of 5 pF was integrated with a small inductor loop to form an oscillating circuit similar in nature to a RFID tag. This formed a passively powered pressure sensor. The power and communication comes from a nearby network analyser, which is connected to another inductor loop, inductively coupled to the sensor. The principle is that a change in pressure will deflect the capacitor changing the resonant frequency of the oscillating circuit. The network analyser, which measures the resonant frequency, can therefore measure the pressure without needing to be directly connected to the sensor, or for the sensor to have its own power source. This means the sensor and associated circuit could be inside the pipe or integrated into the pipe wall, whether it be in the lining or otherwise. For instance, in Figure 16 it can be seen that the frequency of the oscillating circuit changed by 4 MHz when pressure was applied.

**Figure 17.**
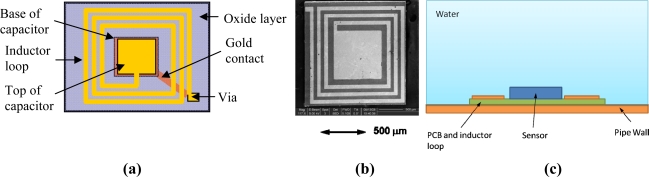
**(a)** Schematic of and **(b)** Scanning Electron Microscope image of the MEMS oscillating circuit [29] and **(c)** potential application on a pipe.

However, the sensor in this circuit was connected to a 5 turn, 10 mm diameter inductor loop with a ferrite core soldered to a surface mount capacitor with a fixed capacitance of 10 pF. This is, of course, too large to be integrated into the pipe wall. Therefore, an investigation into the possibility of miniaturising the sensor and circuit was carried out whereby an oscillating circuit was fabricated using MEMS technology as seen in Figure 17.

**Figure 18.**
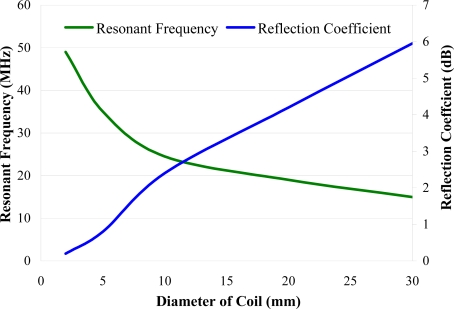
The effect the coil diameter has on the resonant frequency and reflection coefficient (related to signal strength) for a range of simple oscillating circuits.

Initial testing did not detect a signal from the MEMS circuit. Further tests on larger resonator circuits showed that there is a limit to how small an oscillating circuit can be made that is detectable from a reasonable distance. For instance, Figure 18 shows how the signal strength reduced with decreasing inductor loop diameter. This implies that the range over which the circuit can be detected is limited by the coil size using this method. This suggests that either an alternative power supply or communication system may be required which will increase the size of the device. This will be subject of further investigation.

## 4. Discussion and Conclusions

Overall, the results of the smart pipe demonstrator unit burial, and associated tests, are considered to have provided a unique insight into the potential issues associated with incorporating sensors and electronics into buried pipelines. This is the first prototype of its kind which actually attaches a combination of different sensors to a pipe and buries it underground. All these sensors recorded data thus showing that they can survive in a harsh environment although improvements with regards to the ruggedness and miniaturisation of the sensors are required in the future. The following sections give more detailed comments on different aspects of the work.

### 4.1. Communication

Low frequency communications conducted using relatively large inductor loops have shown that communication through water, soil, plastic and air are possible. The range has been shown to be of the order of a few metres using simple equipment and this can be improved with the appropriate use of amplifiers and coil design. The main issue with this method is the size of the coils (which is proportional to the frequency used) required to produce a significant signal; however, it has been shown that a coil of the same diameter as the pipe is sufficient for communication along the pipe through water for a considerable range.

Attenuation of signals increases with frequency, limiting the range of high frequency communications considerably. It has been shown that a signal can still be detected over a couple of metres in water and soil, but the complexity of the circuit required to achieve this is significantly higher than necessary for low frequency communications. If used in air to perhaps communicate between Smart Servers, high frequency communications fair far better and can be used to transmit over a greater distance than low frequency methods using common commercial devices such as Bluetooth.

### 4.2. Smart Server

The Smart Server was successfully used to collect data from various types of sensor and to communicate the results to an operative at ground surface level. It is simple to see how this idea can be expanded to network level where a number of Smart Servers work together to collect and transmit data for a large network of pipes. The server used in this project was a prototype, but it is obvious that it can be further developed and miniaturised using bespoke integrated circuit technology to make it smaller, more efficient and easier to deploy.

### 4.3. Sensors

Several commercial MEMS type sensors were used to help demonstrate the feasibility of the smart pipe concept. The piezoelectric transducers, the force sensor and the accelerometer were sensitive to different excitations. For example, the piezoelectric transducers reacted well to excitations from a revving car at the ground surface and thus suggesting that they may not only be used as sensors, but are also sensitive enough to be used as an energy scavenging device. The laser module detected large obstructions as well as the attenuation caused by different media (air/water). This suggests that in the future, the turbidity in the water could be measured using such a device. The temperature sensors also demonstrated how multiple sensors could be used to monitor discrete parameters, in this case the temperature of the water. Although most of these sensors would need modification for a commerical smart pipes system, for example they are currently too large, they did demonstrate successfully the monitoring possibilities that could be employed and that they could all detect changes in parameters likely to be indicative of a pipe failure (potential leak or overstressing of the pipe). With relevant trigger values put on the sensors outputs it would be possible to have a system that could report potential problems within a pipeline even with these simple sensors.

In parallel, a passive purpose-built sensor using MEMS technology was investigated. This pressure sensor successfully detected changes in pressure using a capacitive circuit. However, using this sensor in a passive mode meant that the range of communication of the data was limited and highly dependent on the size of the inductive loop used. Passive systems have a near-infinite life and require no internal power source, but they offer limited functionality and can be interrogated over only comparatively short ranges, perhaps just a few centimetres. For this reason, active systems, which can offer much great functionality, e.g., basic data processing, and the ability to transmit over significant distances, perhaps many metres, are much more attractive. They do, however, require a reliable power source suitable for use with miniature MEMS systems when embedded in pipe walls, and this presents major technical challenges at this time.

### 4.4. Future Research

Although the smart pipe demonstrator unit described in this paper and the associated research has provided valuable information on producing smart pipes, there are certain areas that need further research if this concept is to become reality. These include:

- Powering the sensors—methods of harvesting energy from the surrounding environment are essential to power the sensors for the lifetime of the pipe. This is particularly important if Smart Pigs for data collection are to be avoided within the water distribution network.
- Communications—further work on sensor-to-sensor and sensor-to-Smart Server communication is required.

Research into these components is currently under way at the University of Birmingham and results will be presented in future papers.

## Figures and Tables

**Table 1.** Signal frequencies and their potential uses [29].

**Signal frequency**	**Potential uses and issues**
Low (kHz)	Communications from sensors in soil, pipe-to-pipe data transfer, long-distance data transfer along pipelines. Low power requirements but low data transfer rate.
High (MHz)	Communications from sensors built into pipes, short range data transfer along pipelines, data transfer to surface in chambers. Can communicate more detailed data at higher rates but high power consumption.
